# Supplementary material for: Structures of a deAMPylation complex rationalise the switch between antagonistic catalytic activities of FICD
Source: Nat Commun. 2021 Aug 18;12:5004. doi: 10.1038/s41467-021-25076-7 (PMC8373988; doi:10.1038/s41467-021-25076-7)
Supplement: Supplementary file 1 — Supplementary Information [file 41467_2021_25076_MOESM1_ESM.pdf]

Supplementary Information

Structures of a deAMPylation complex rationalise the switch between antagonistic catalytic activities of FICD

Luke A. Perera*, Steffen Preissler, Nathan R Zaccai, Sylvain Prévost, Juliette M Devos, Michael Haertlein, David Ron*.

PAGE DELIBERATELY BLANK

Supplementary Fig. 1

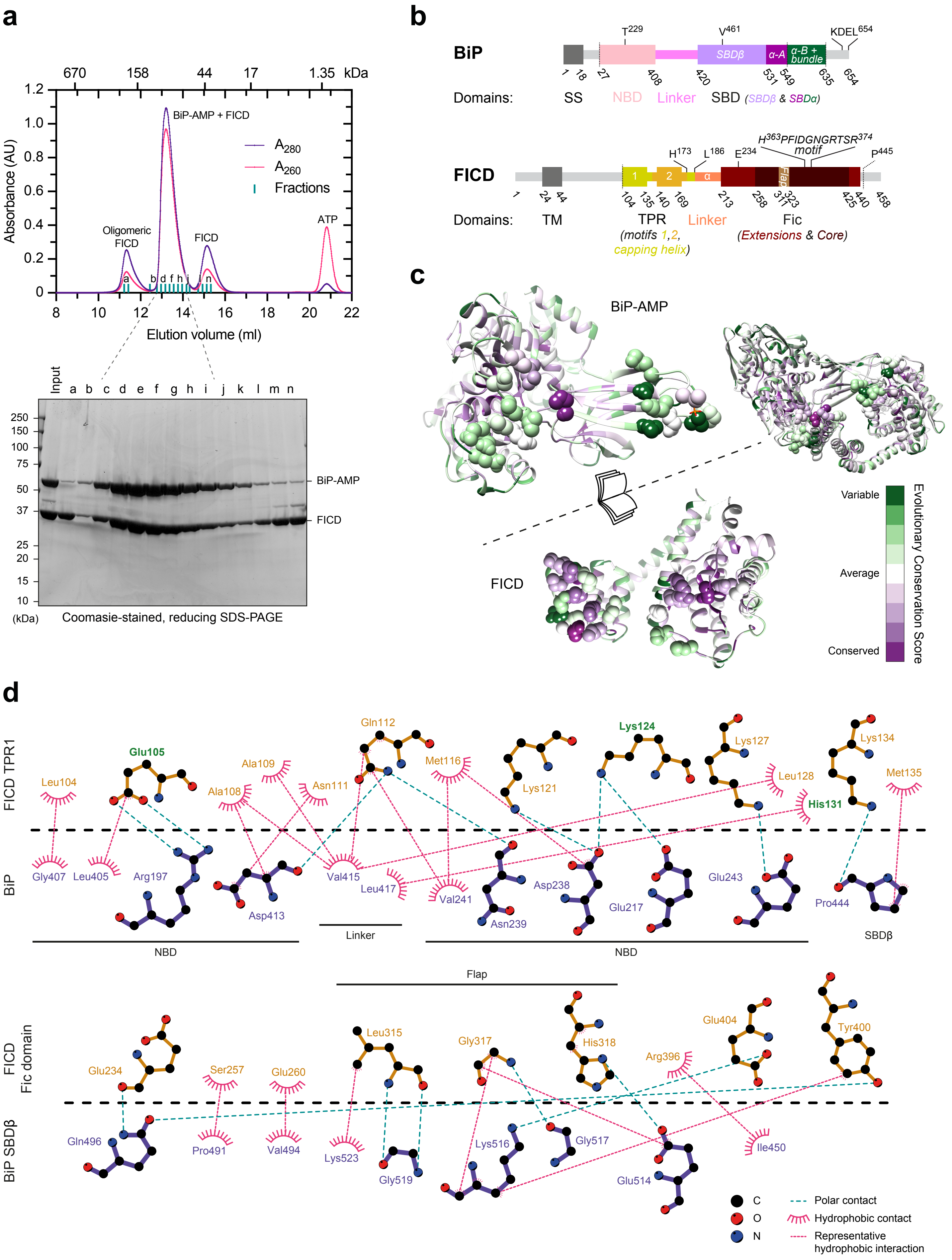

Supplementary Fig. 1: Schematised view of FICD•BiP-AMP intermolecular contacts. a, FICD's His363Ala mutation facilitates trapping and SEC-based copurification, of a deAMPylation complex of monomeric FICD and AMPylated BiP. Shown is a representative chromatogram from n = 2 independent purifications. **b,** Schematic representation of Chinese hamster BiP and human FICD domain organisation (SS; signal sequence; TM, single-pass transmembrane domain). Various sub-domain features are also annotated in italics (SBDα-A/B, SBDα helix A/B; Bundle, SBDα's α-helical bundle). Light grey bars represent predicted unstructured regions. Recombinantly expressed and purified BiP and FICD constructs utilised in this study encompass residues 27–549 and 27–635 of BiP (for crystallographic and biochemical purposes, respectively) and residues 104–445 of FICD (vertical dashed lines). Note, the amino acid sequence is identical between human and hamster BiP between residues 27–635. All regions are coloured according to the scheme used in **Fig. 1** (with the exception of the core Fic domain which, in **Fig. 1**, is not coloured separately from the N- and C-terminal Fic domain extensions). **c,** Structures of isolated BiP-AMP (PDB 5O4P, chain B) and FICD (PDB 4U04, chain B) coloured (per residue) by degree of evolutionary conservation amongst Fic and Hsp70 proteins, respectively. The structures are portrayed with an 'opened' deAMPylation complex interaction interface and residues observed to be involved in intermolecular contacts within the complex are depicted as spheres. Top right, the 'closed' complex of FICD and BiP is shown aligned to the FICD•BiP-AMP deAMPylation complex (grey). Interacting residues appear more conserved across FICD, where interacting residues are either located within the TPR domain (which is specific to metazoan FICD homologues) or close to the nucleotide binding pocket of the Fic domain. Likewise, the BiP NBD residues that interact with FICD(TPR) are conserved across metazoan BiP homologues (see sequence alignment in[1]). **d,** All polar (hydrogen bonds and salt bridges) and hydrophobic protein-protein contacts between FICD and BiP in the deAMPylation complex crystal structures are illustrated. The (sub)domain origin of the interacting residues are also annotated. Residues mutated in the study are labelled in green.

Supplementary Fig. 2

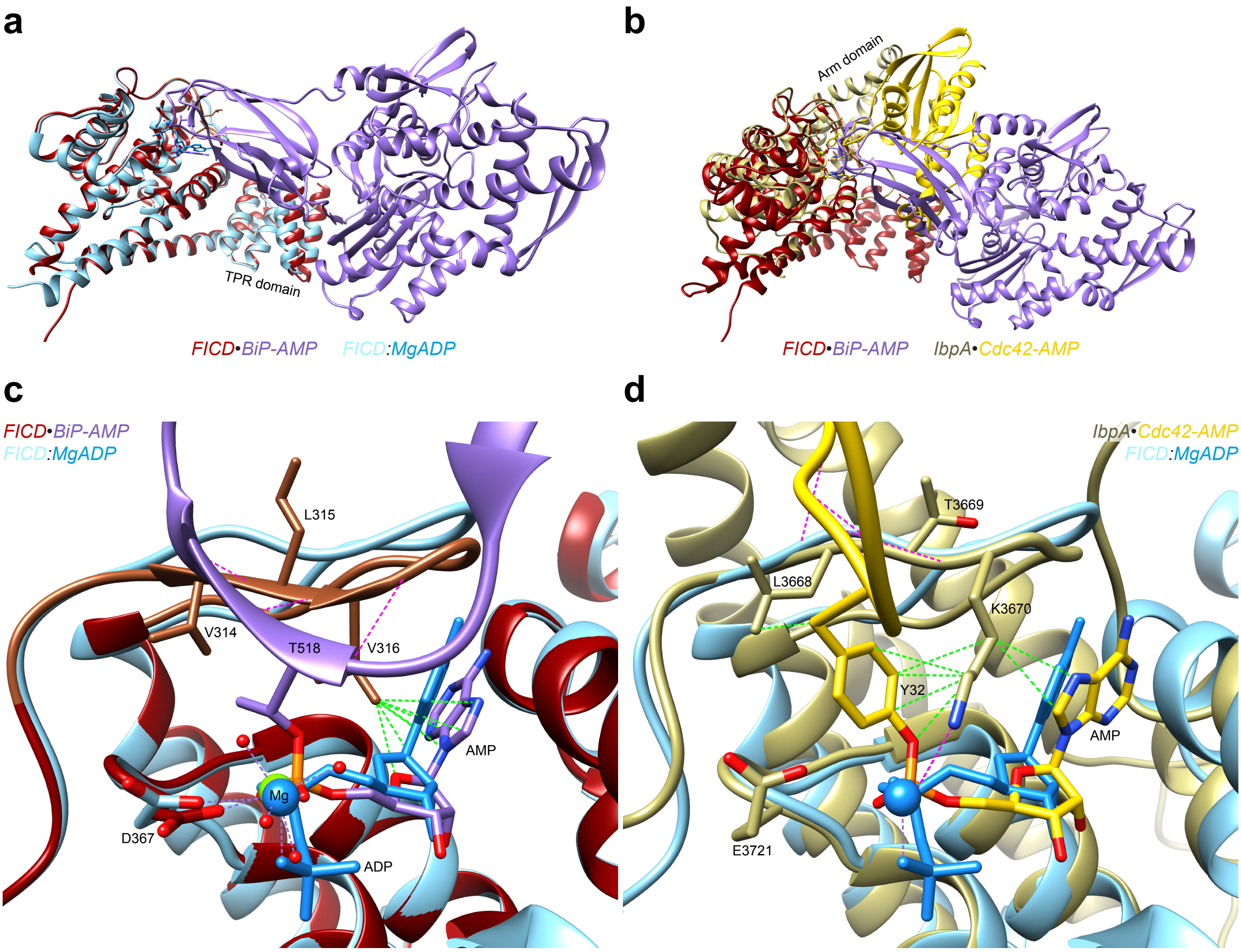

Supplementary Fig. 2: Comparison of modes of target-residue recognition by FICD and IbpA. a–b, Structural superpositions of the state 1 deAMPylation complex (FICD•BiP-AMP) with either, **a**, a single protomer of the dimeric FICD:MgADP (PDB 4U0U) structure or, **b**, the IbpA•Cdc42-AMP post-AMPylation complex (PDB 4ITR). All structures are aligned by residues 348–384 of FICD (which encompasses the conserved Fic motif). The target protein recognition-specific modules of both Fic proteins are also annotated (TPR and arm domain, respectively). **c–d,** More focussed views of the Fic domain flap-substrate interacting regions present within the deAMPylation complex (**c**) and IbpA•Cdc42-AMP (**d**). The FICD:MgADP structure is shown for reference, and in the same orientation, in both panels. Hydrogen bonds and hydrophobic interactions between the AMPylated target residue and the Fic domain flap residues adjacent to the target residue are indicated in each protein complex (pink and green dashed lines, respectively). In **c** the side chains of residues Val314 and Val316, which are located within the Fic domain flap of FICD (residues 311–323, highlighted in brown) and sit either side of BiP(Thr518-AMP), are not within hydrophobic interaction distance of the Thr518 side chain. In contrast, in **d**, the Fic flap of IbpA (via Leu3668 and Lys3670) hydrophobically clamps the Tyr32 target residue of Cdc42 (as annotated). Note, both FICD's Val316 and, to a lesser extent, IbpA's Lys3670 form hydrophobic contacts with the adenosine ring.

Supplementary Fig. 3

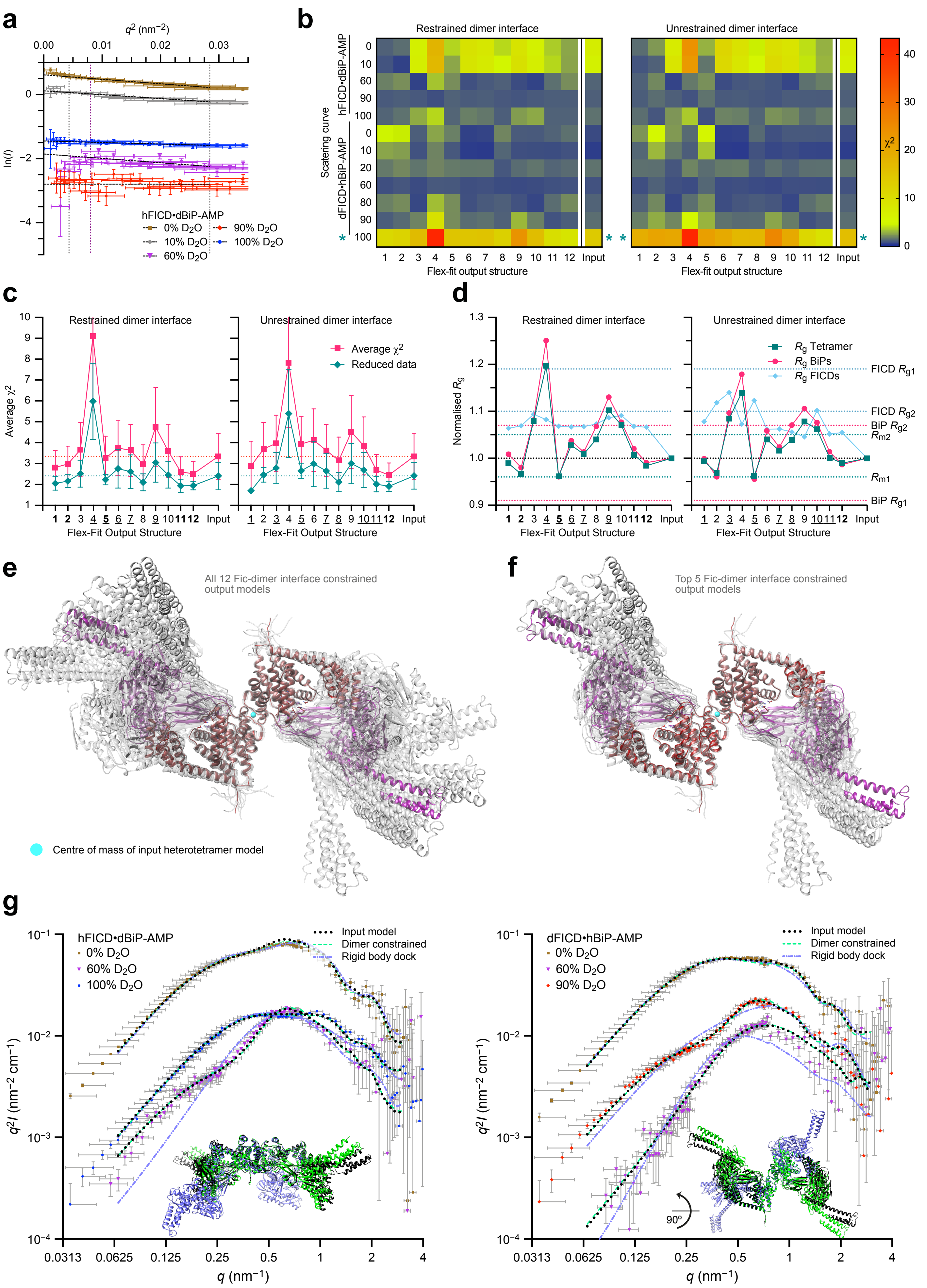

Supplementary Fig. 3: SANS data analysis. a, Guinier plot of non-deuterated FICD with partially deuterated AMPylated BiP (derived from the scattering data shown in **Fig. 2a**, with mean ± SEM values plotted). The *q*-range for fitting is denoted as in **Fig. 2b** with the exception that the low-*q* limit for the scattering of hFICD.dBiP-AMP in 60% D_2O is represented by the vertical, purple dotted line. **b,** Heat-map of the χ^2 goodness of fit of the theoretical scattering curve of each flex-fit model against all observed experimental scattering datasets. The major diagonal (top left to bottom right) illustrates the optimised χ^2 of each flex-fit model from its progenitor dataset. **c,** Comparison of the mean χ^2 for each model derived from analysis of the goodness of fit to all scattering datasets (generated from both oppositely labelled complexes). The 'reduced data' average χ^2 (green) is derived from fitting to all data excluding the anomalous scattering observed for dFICD•hBiP-AMP in 100% D_2O (* in **b**). Error bars represent standard errors of the mean (SEM) of each average χ^2 value (n = 12 and n = 11 for the complete and reduced scattering datasets, respectively) and the horizontal dotted lines illustrate the χ^2 values of the input model. **d,** Comparison of Stuhrmann analysis derived R_gs (horizontal dotted lines) with the calculated R_gs of the input and output structures. In **c** and **d** output structures highlighted in bold have χ^2 variances (for reduced data) which are less than and significantly different to the input model's χ^2 variance ($P < 0.05$ by F test); symmetrical output structures are underlined. **e,** Superposition of all 12 flex-fit output structures (with FICD dimer interface constrained) relative to the input heterotetramer model (red FICD dimer, purple BiP-AMPs). **f,** As in **e** but only displaying the top 5 flex-fit structures with significantly reduced χ^2 SDs. **g,** Kratky plots of representative scattering curves highlighting the relative fits of the input, dimer constrained best-fit and a poorer fitting rigid-body docking models. Mean values are plotted ± SEM with respect to the number of pixels used in the radial data averaging. Inset, colour-matched structures aligned by the FICD dimer, shown in orthogonal views. Note, the scattering intensity profiles are consistent with FICD•BiP-AMP being a folded protein complex. See **Supplementary Movie 1**. Source data are provided as a Source Data file.

Supplementary Fig. 4

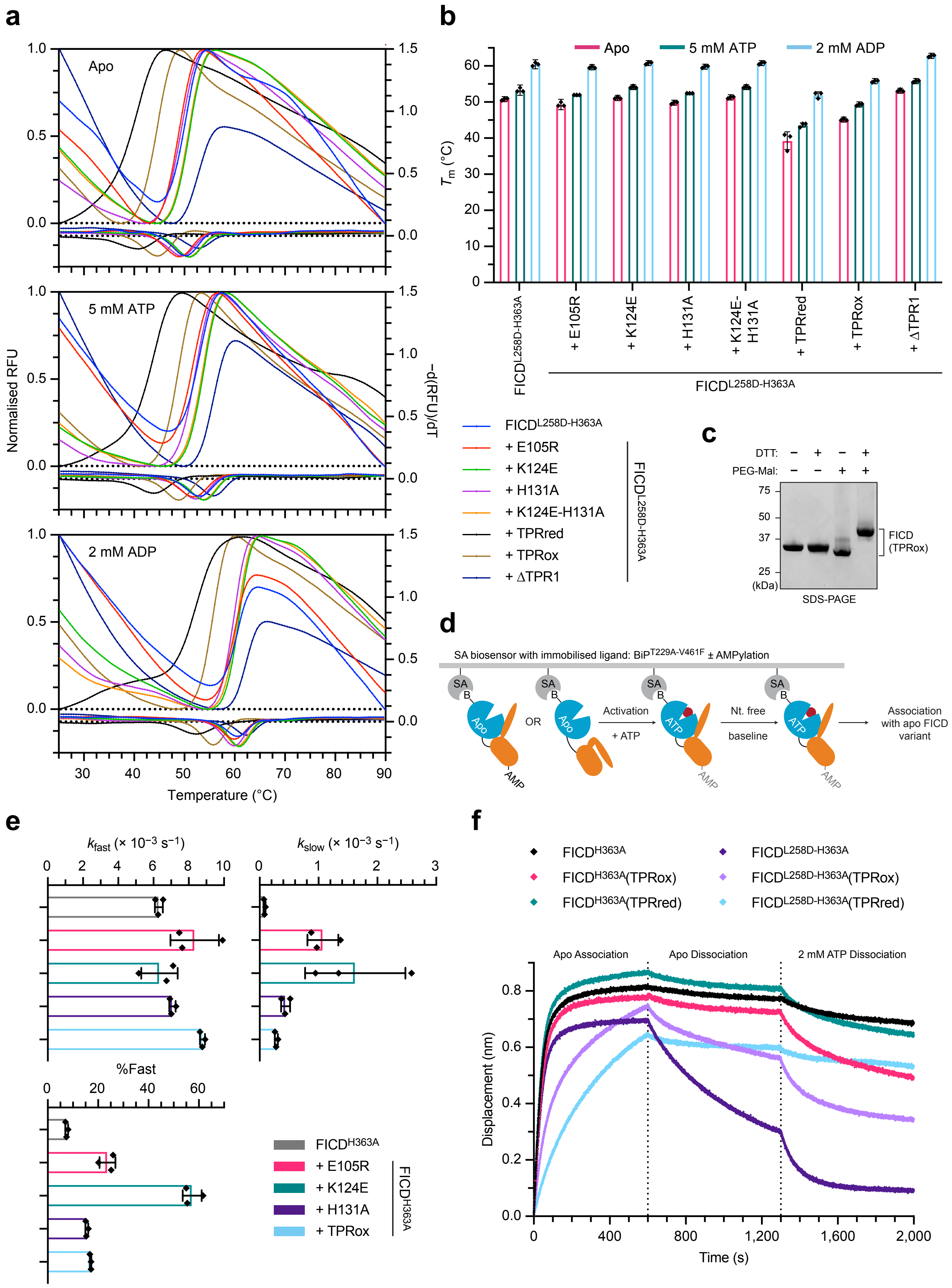

Supplementary Fig. 4: Biophysical analysis of FICD mutants and their binding to AMPylated BiP. a, Representative normalised DSF melt curves (top curves) with corresponding negative first derivatives (bottom curves). **b,** The derived protein melting temperatures (T_m, mean ± 95% confidence interval (CI)) derived from n = 3 independent DSF experiments each conducted in technical duplicate. Note, all FICD variants (like FICD$^{L258D-H363A}$) are stabilised by nucleotide binding. **c,** PEG 2000 maleimide-based electrophoretic mobility assay analysis of the oxidation status of monomeric FICD$^{L258D-H363A}$(TPRox), demonstrating nearly complete disulphide-stapling of the FICD's TPR domain to its linker helix. The gel is representative of n = 3 independent experiments. **d,** Schema of the BLI steps preceding the association and dissociation phases of FICD with immobilised BiP (± AMP) shown in **Fig. 3a–b**. See **Supplementary Note 1**. **e,** The kinetic parameters of the 2-phase dissociation of dimeric FICD variants (in the presence of excess ATP) as derived from the representative fitting in **Fig. 3b*(ii)***. Mutation of the TPR domain accelerates FICD dissociation from BiP:ATP, notably increasing the slower dissociation rate constant (k_{slow}) and the percentage of the biphasic dissociation attributed to the fast phase (%Fast). Mean values ± SD are shown, for each kinetic parameter, from n = 3 independent experiments. **f,** BLI analysis of BiP-AMP binding by reduced (TPRred) and oxidised (TPRox) monomeric FICD$^{H363A-L258D}$ and dimeric FICD^{H363A} both mutated at Asp160Cys, Thr183Cys and Cys421Ser. Note the dimeric forms of TPRred and TPRox behave similarly whilst the monomeric forms of FICD TPRox and TPRred exhibit distinct binding behaviour. The latter associates slowly and irreversibly with BiP-AMP, consistent with non-specific binding. The irreversibly binding is likely a consequence of protein instability at the temperature at which the BLI experiment is conducted (30˚C, close to the melting temperature of the monomeric TPRred, see **a & b** above). Source data are provided as a Source Data file.

Supplementary Fig. 5

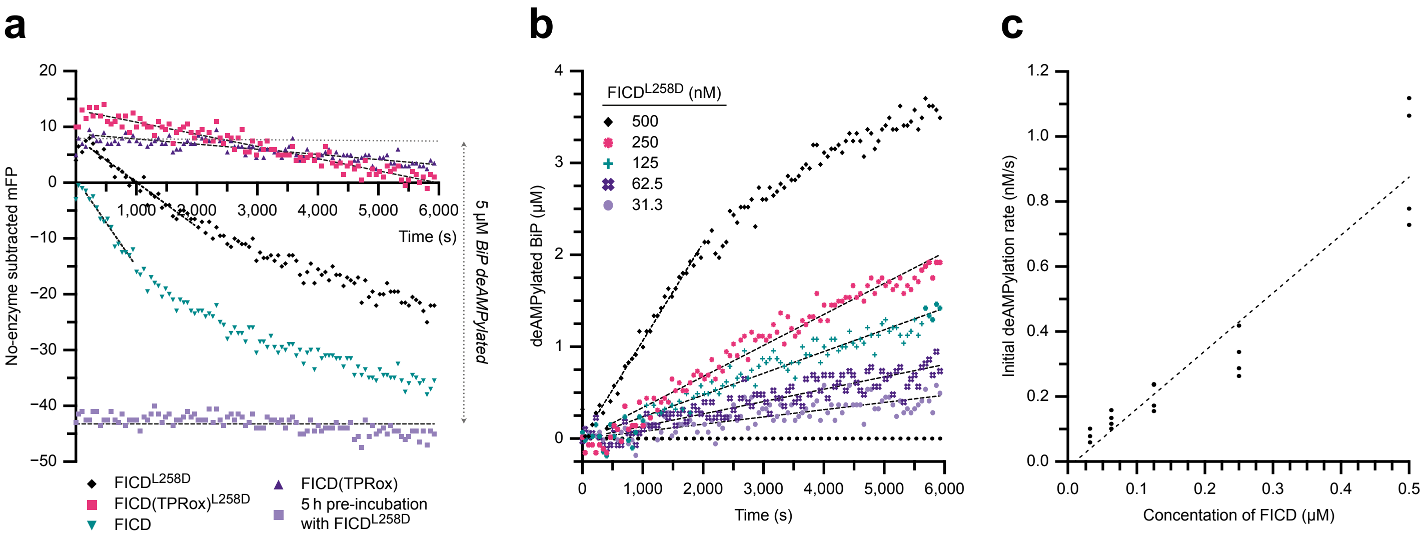

Supplementary Fig. 5: In vitro deAMPylation assay data processing and assay validation. **a,** The FP curves from which **Fig. 3c** (lower panel) was derived. The difference (in mFP units) at t = 0 between the FICD^{L258D} deAMPylation time course and the pre-incubated and fully deAMPylated reaction was taken to represent complete substrate deAMPylation (5 µM BiP-AMP). Fits over the initial linear range of the reaction are overlaid. Fitting was conducted over the initial time range where the enzyme velocity is approximately constant and is thus representative of v_0 (see **Methods**). **b,** A time course of BiP-AMP(FAM) deAMPylation with different concentrations of FICD^{L258D}. **c,** Quantification of the assay represented in **b**, from n = 4 independent experiments, demonstrating the minimum linear dynamic range of the assay. The dashed line illustrates the determined best-fit linear relationship. Note, no fitting constraints were placed on the gradient or *y*-intercept parameters. Source data are provided as a Source Data file.

Supplementary Fig. 6

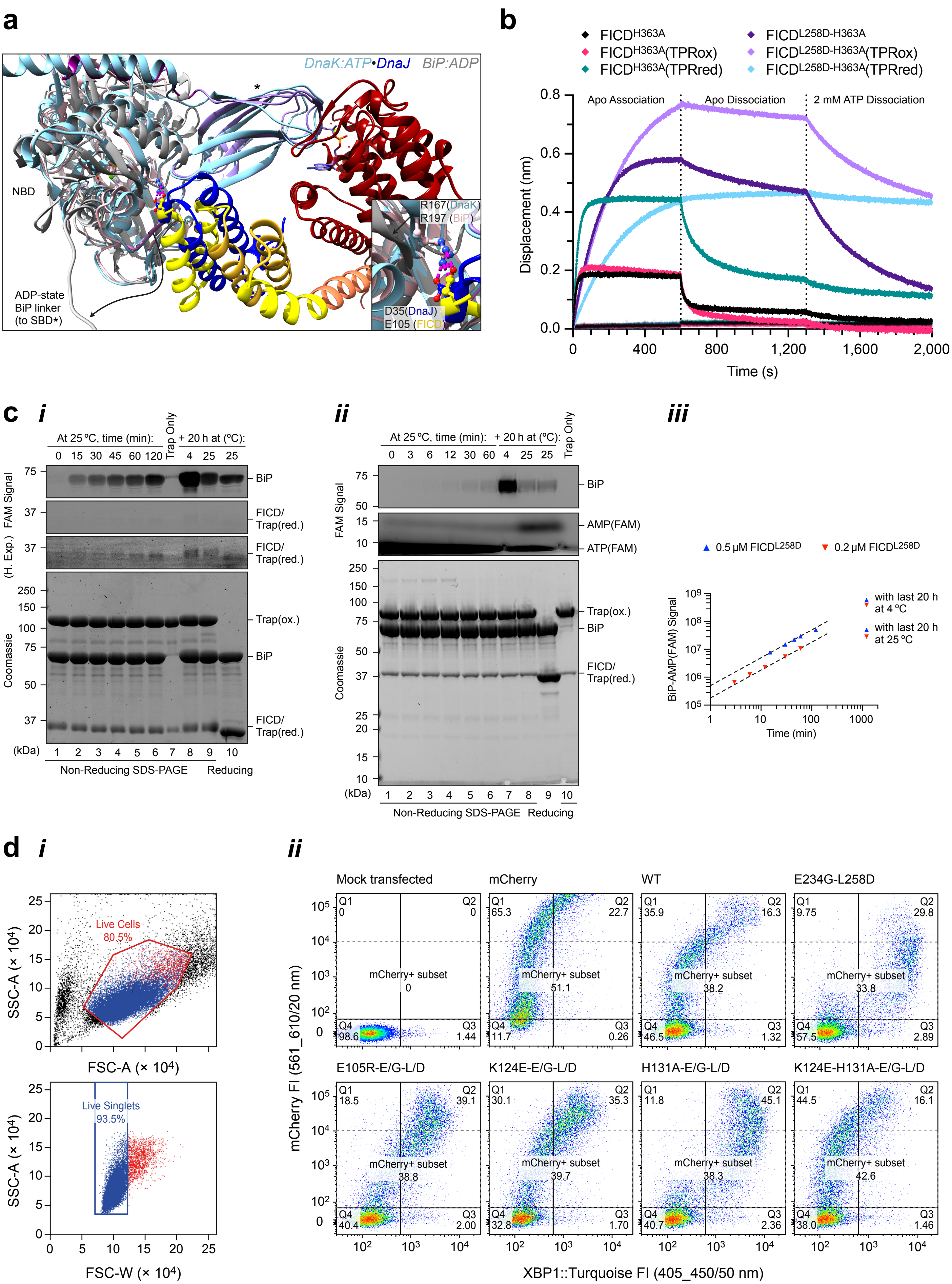

Supplementary Fig. 6: FICD recognises the ATP-state of BiP. a, FICD's TPR domain and the J-domain recognise similar ATP state–specific Hsp70 surfaces. A structural superposition of the deAMPylation complex and ADP-state BiP (as in **Fig. 4a*(i)***) along with the structure of DnaK:ATP bound to DnaJ (PDB 5NRO, light and dark blue, respectively). All structures are aligned via their Hsp70 NBDs. The arrow indicates the displacement of the linker from the BiP ATP to ADP-state. Inset, the conserved DnaJ HPD-motif contact (DnaK Arg167 to DnaJ Asp35) is also mimicked in the BiP(NBD) to FICD(TPR1) interaction (Arg197 to Glu105, respectively). **b,** The effect of cysteine oxidation on FICD binding to immobilised BiP:ATP assayed by BLI. Intramolecular disulphide bond formation significantly affects the binding of both monomeric and dimeric FICD variants. Interestingly, oxidation appears to strengthen monomer binding and, conversely, inhibit FICD dimer binding to BiP:ATP. Note, $FICD^{L258D-H363A}$(TPRred) binds completely irreversibly to BiP:ATP (consistent with its thermal instability, see **Supplementary Fig. 4a–b** and **f**). Semi-translucent colour matched BLI traces, at < 0.02 nm displacement, indicate the binding of each FICD variant to the biosensor alone. **c,** $FICD^{L258D}$ BiP-AMPylation time courses (experimental design as in **Fig. 5a**) with quantification of two independent experiments in ***(iii)*** (dashed lines; directly proportional fits). In ***(i)*** the concentration of $FICD^{L258D}$ was 0.5 μM and proteins were resolved by 10% SDS-PAGE, as in **Fig. 5a**. In ***(ii)*** 0.2 μM $FICD^{L258D}$ was utilised, and the proteins were resolved by a 4–12% gradient SDS-PAGE. Note, the difference in BiP AMPylation between overnight incubation of the AMPylation reaction at 4 and 20 °C. This reflects the increased rate of BiP-AMP escape from the trap and deAMPylation, as evidenced by the conspicuous increase in free AMP(FAM) nucleotide analogue resolved in ***(ii)***. **d,** FACS dot-plots. ***(i)*** A representative gating strategy for the selection of live, single cells (from forward/side scatter (F/SSC) area/width (A/W) measurements), which were further assessed as in ***(ii)***. Cells with mCherry+ signal $\leq 10^4$ were gated, eliminating the distorting effect of high FICD expression (marked by the mCherry) on the XBP1::Turquoise signal, and further analysed in **Fig. 6c**. Source data are provided as a Source Data file.

Supplementary Fig. 7

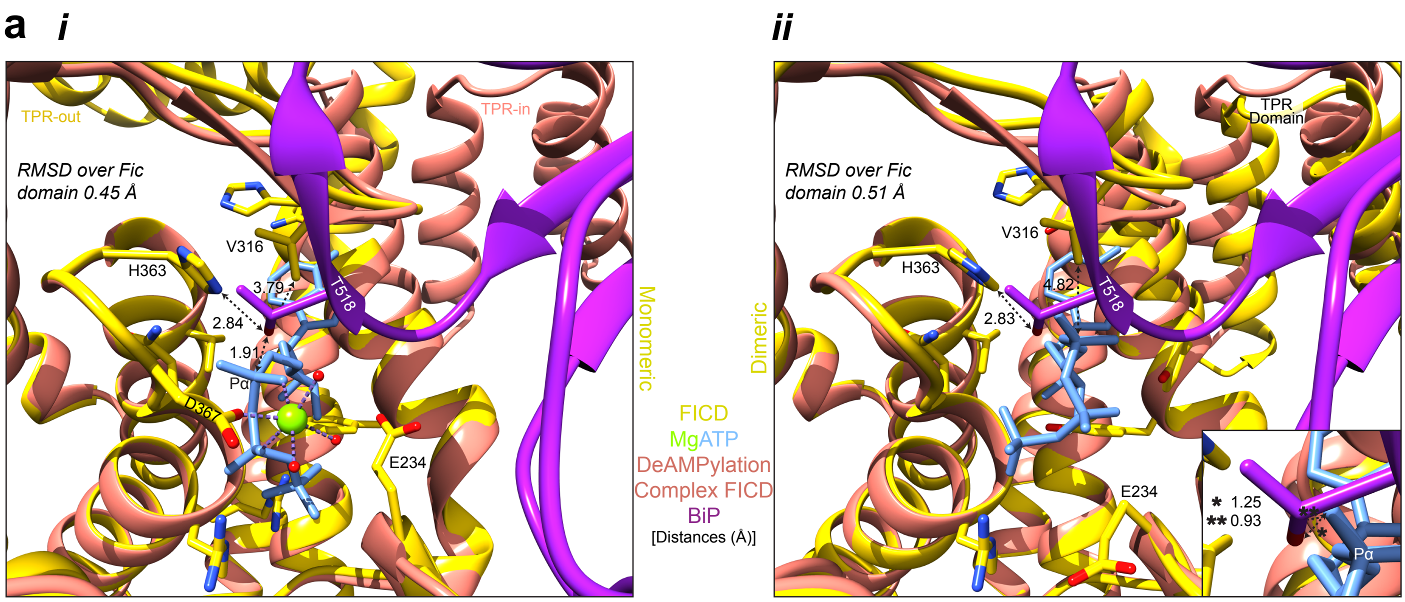

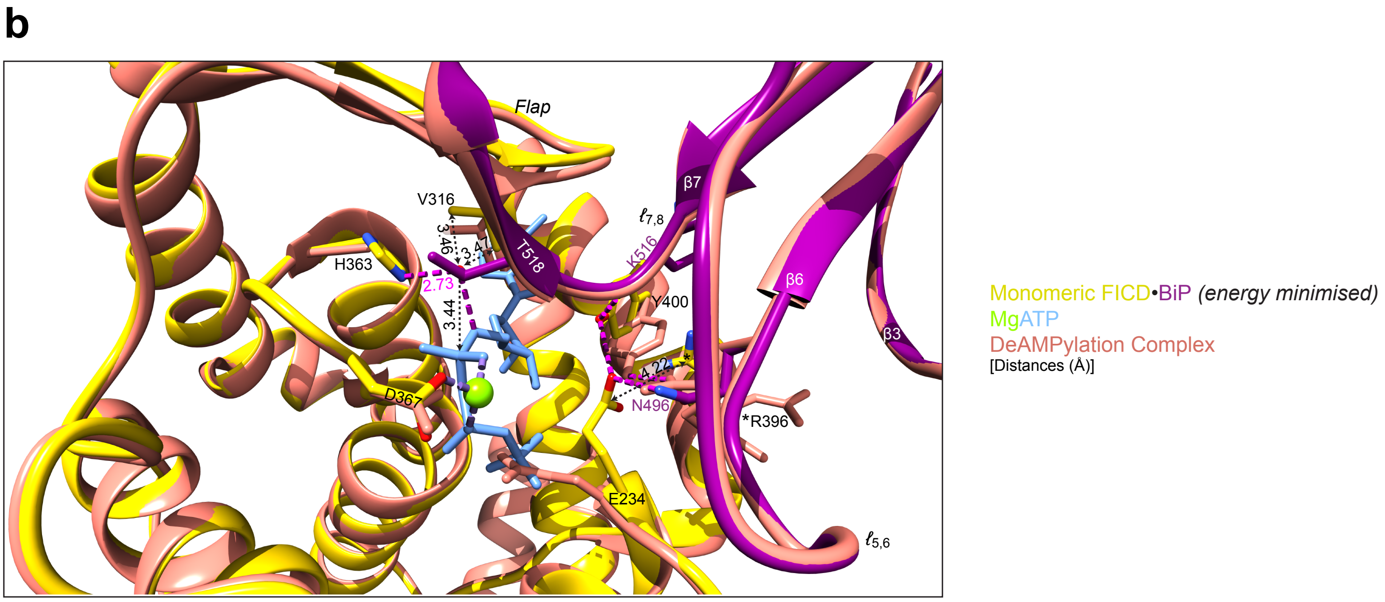

Supplementary Fig. 7: Predicted structure of the AMPylation complex active site. a, (*i*) Structural superposition illustrating the ability of monomeric FICD, bound to MgATP (PDB 6I7K; yellow with purple nucleotide), to accommodate BiP's Thr518 in a catalytically-competent conformation. The superposition is derived by alignment of FICD:MgATP with FICD from the deAMPylation complex (orange; BiP in purple). Oγ of BiP's Thr518 (restored by removal of the AMP) is in-line with the Pα-$O^{3\alpha}$-phosphoanhydride bond and can be deprotonated by His363. Though not modelled here, flexibility in the Fic flap, FICD's Val316 and BiP($\ell_{7,8}$) likely permit Pα and Oγ(Thr518) to attain a distance consistent with an initial substrate engagement state. **(*ii*)** As in ***(i)*** but with alignment of dimeric FICD bound to ATP in a catalytically-incompetent mode (PDB 6I7G). Note the severe clash between Thr518 and the ATP α-phosphate (*inset). **b,** A putative AMPylation complex active site (purple 'unmodified' BiP from the deAMPylation complex and yellow $FICD^{L258D}$:MgATP) was modelled by energy-minimisation of the structural alignment shown in **a*(i)***. The derived local energy minimum (FICD•BiP with MgATP) is also superposed with the original state 1 deAMPylation complex (FICD•BiP-AMP, orange, with the AMP moiety removed). Selected intermolecular hydrogen bonds (pink dashed lines) and interatomic distances (black dashed lines) are annotated. Note, unmodified BiP is able to form an intermolecular β-sheet with the flap of $FICD^{L258D}$ whilst positioning its target reside (Thr518) in-line for nucleophilic attack into the ATP α-phosphate, within hydrogen bonding distance of the general base His363 and without any steric clashes. Glu234 may form additional direct contacts with BiP via a salt bridge-stabilised, intermolecular hydrogen bond network unique to the AMPylation complex. Minimisation was carried out with the ATP and FICD's Arg374 fixed in their original (FICD:MgATP structure) position. A portion of both FICDs' α-helical linkers and the complete TPR domains (visible in the background of **a**) are omitted for clarity.

Supplementary Fig. 8

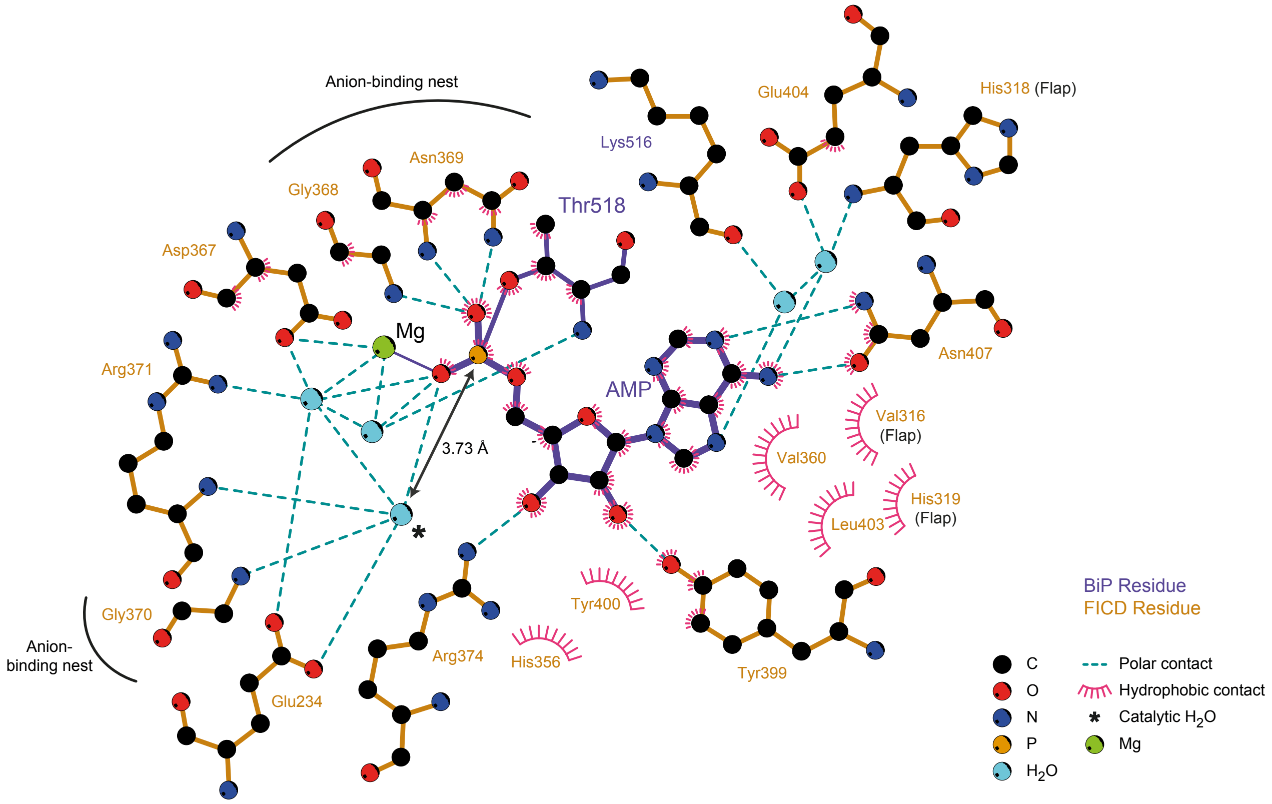

Supplementary Fig. 8: Interactions of BiP's Thr518-AMP within the active site of FICD. Intermolecular contacts formed by BiP's Thr518-AMP and complexed Mg^{2+}, as shown in **Fig. 6a**, are depicted. Various Fic domain features and the distance between the catalytic water* and the AMP phosphorous atom (green sphere) are also annotated. Note the tight binding of the adenosine and α-phosphate within the catalytic Fic domain. Coordination of the ribose 3'OH by FICD's Arg374 would prohibit the intramolecular nucleophilic attack and cyclisation required for an anchimeric-assisted mechanism of BiP deAMPylation (an alternative mechanism capable of generating the experimentally-observed deAMPylation products, unmodified BiP and AMP[2]). Likewise, the lack of a base (required for proton abstraction) in the vicinity of BiP's Thr518 Cα speaks against an E1cB-type elimination-based deAMPylation reaction.

Supplementary Fig. 9

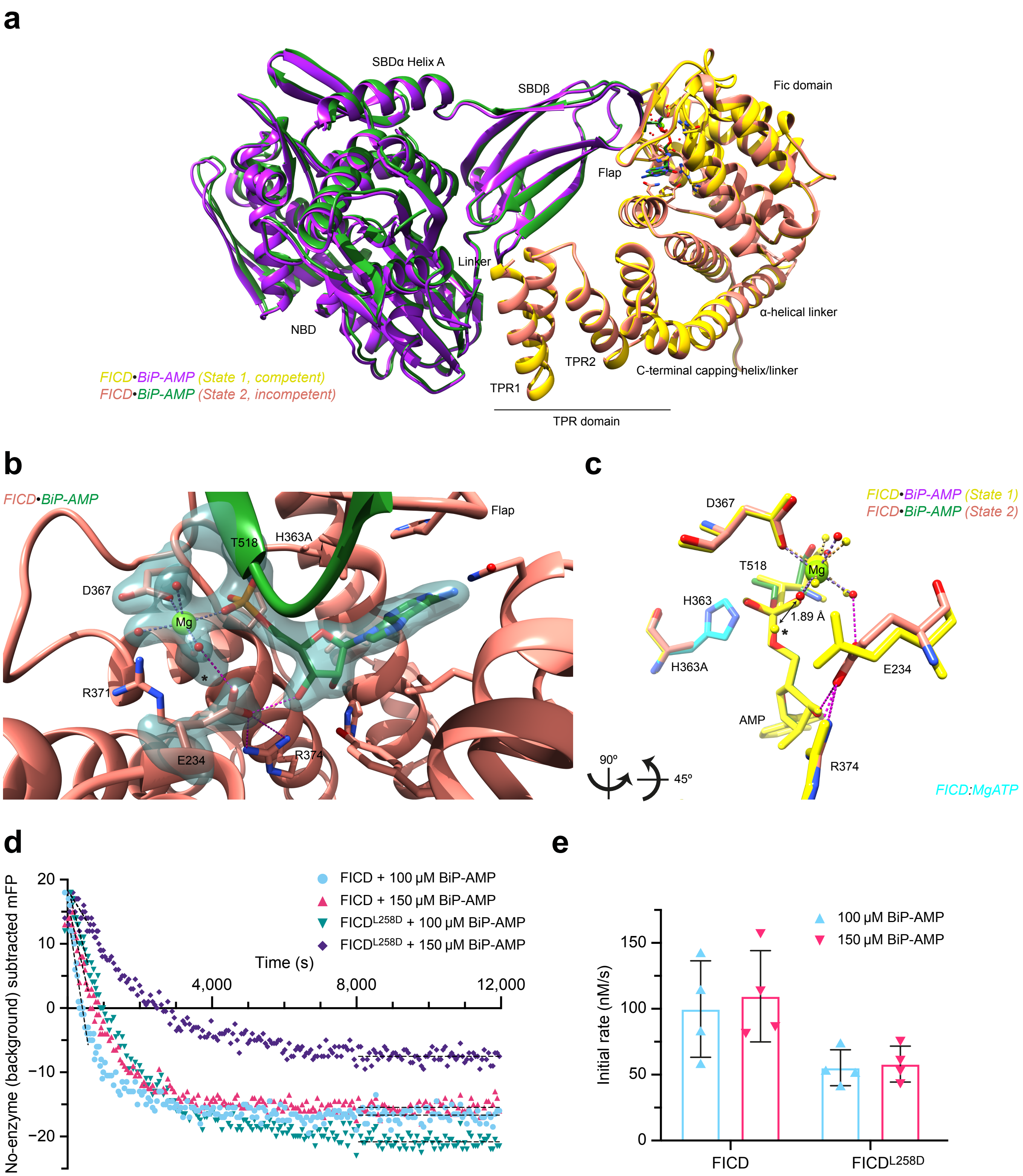

Supplementary Fig. 9: A second deAMPylation complex crystal structure captures a non-catalytically competent state of monomeric FICD. a, Superposition of state 1 and state 2 heterodimeric deAMPylation complexes, illustrating the high degree of overall similarity between the two. See **Supplementary Movie 1**. **b,** A focus on the state 2 deAMPylation complex active site, with regions of particular interest additionally overlaid with an unbiased polder OMIT electron density map, contoured at 6σ. See **Supplementary Movie 3**. In **a** and **b** structures are depicted in the same view as the state 1 complex shown in **Fig. 1a** and **Fig. 6a**, respectively. **c,** The same reduced active site view as shown in **Fig. 7a**, with the polder OMIT map removed for clarity. In **b** and **c** interactions formed by state 2's Glu234 are shown with pink-dashed lines. See **Supplementary Movie 3**. **d,** Background drift–subtracted FP deAMPylation time course, the basis of the panel displayed in **Fig. 7b**. Linear best-fits are overlaid illustrating the initial reaction progress and final plateau value, the ΔFP between y_0 and y_∞ was taken to represent $[\text{BiP-AMP}]_0$. **e,** Quantification of the initial deAMPylation rates (mean ± SD) with either 100 or 150 µM BiP-AMP substrate at t = 0. Results are presented from n = 4 independent experiments. Source data are provided as a Source Data file.

Supplementary Fig. 10

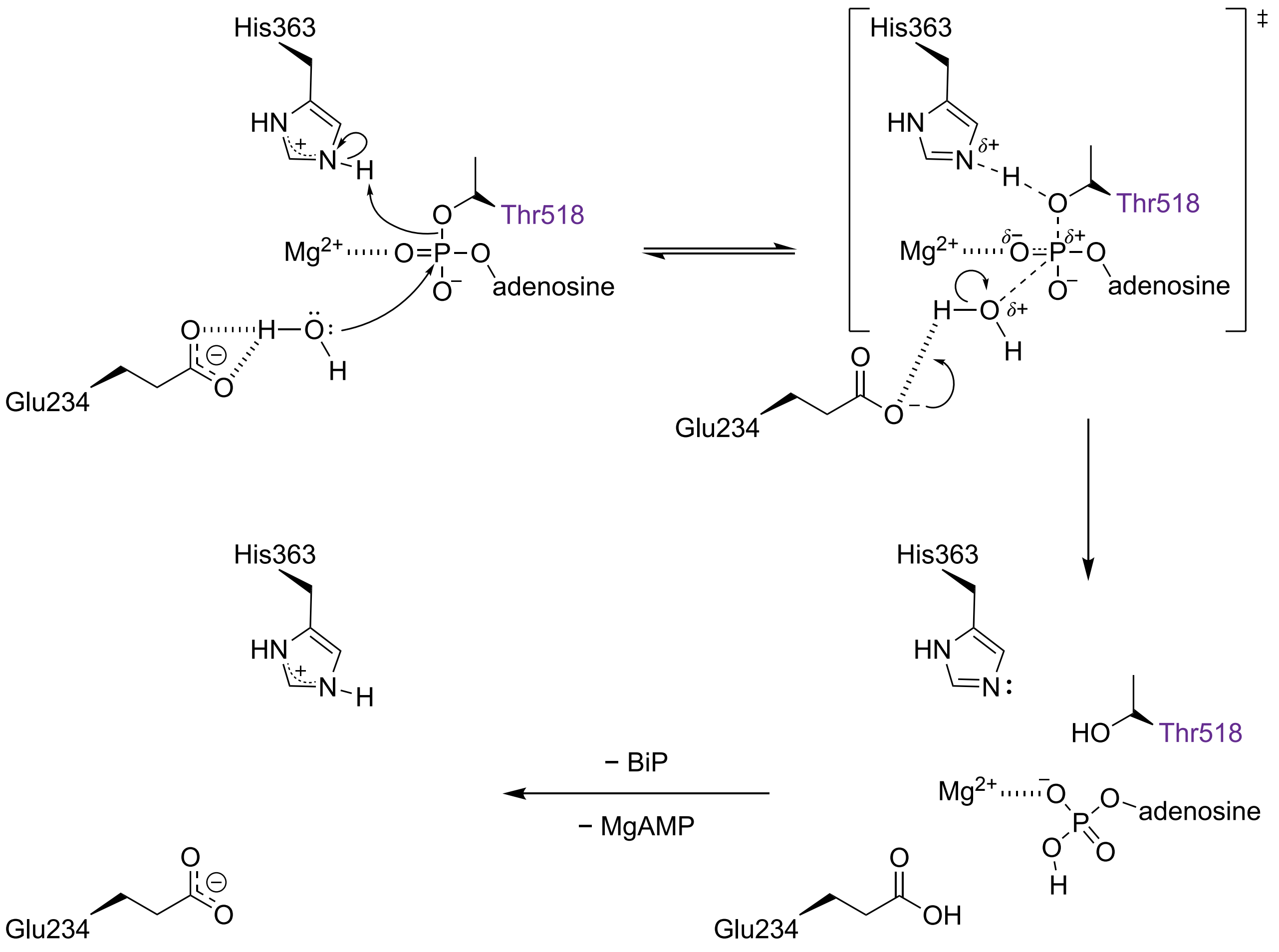

Supplementary Fig. 10: Proposed hydrolytic BiP deAMPylation mechanism. As in the state 1 deAMPylation complex structure, FICD's Glu234 activates and aligns a catalytic water molecule for in-line nucleophilic attack into the backside of AMPylated BiP's Pα-Oγ(Thr518) phosphodiester bond. The correct positioning of Glu234 also facilitates the localisation of the Mg^{2+}-primary hydration sphere in a position compatible with an aligned catalytic water (**Fig. 7a**). The α-phosphate group's coordination by Mg^{2+}, and localisation within FICD's electron withdrawing anion-binding nest (**Fig. 6a** and **Supplementary Fig. 8**), stabilises the position of Pα and increases its electrophilicity. His363 can exist in either a protonated or deprotonated state. The former is required for deAMPylation and is shown. The reaction likely proceeds via a nucleophilic S_N2-type pathway with concerted protonation of BiP's Thr518 alkoxide leaving group (catalysed by FICD's His363 acting as a general acid). A potential role for FICD's Glu234 acting as a catalytic (but not general) base, accepting a proton from the nucleophilic water at a late stage of the reaction after formation of the pentacoordinate transition state (‡), is shown. Glu234 not acting as a general base (but potentially instead as a late-stage proton trap) is consistent with its interaction with Fic motif Arg371 and Arg374 (not shown; see **Fig. 6**), which will depress its pK_a. The schematised hydrolytic reaction generates BiP (with an unmodified Thr518) and AMP. Following product release Glu234 and His363 could facilely exchange protons with the solvent to regenerate the original FICD active site. Polar interactions are denoted with hashed lines, dashed lines represent partial covalent bonds and partial charges are indicated by δ. BiP's Thr518 residue is annotated in purple.

Supplementary Fig. 11

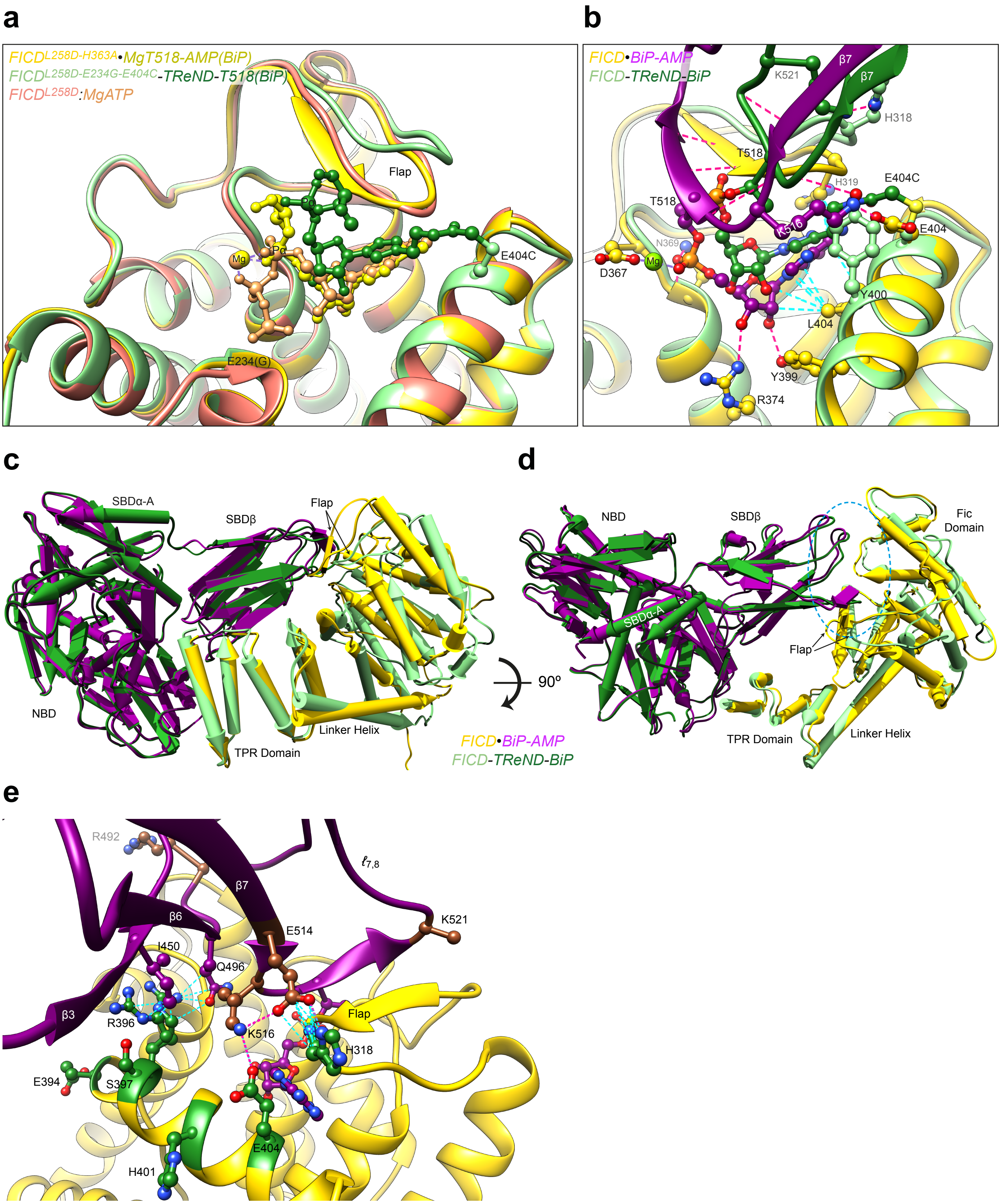

Supplementary Fig. 11: Comparison of the deAMPylation complex with a covalent complex of FICD and BiP. a, Structural superposition of the FICD catalytic domain (complete with active site bound ligands) from the state 1 deAMPylation complex ($FICD^{L258D-H363A}$•BiP-AMP; yellow), a complex of FICD and BiP covalently stabilised by a linking thiol reacted nucleotide derivative (TReND; $FICD^{L258D-E234G-E404C}$-TReND-BiP; PDB 6ZMD; green)[1] and $FICD^{L258D}$:MgATP (PDB 6I7K; orange)[3]. Note, although the TReND is covalently attached to FICD's Glu404Cys, its triazole ring, ribose and α-phosphate (Pα) are all displaced from their canonical position in FICD's nucleotide binding site and its α-phosphate is not coordinated by Mg^{2+}. Contacts formed by adenosine of the deAMPylation complex but not by the TReND are further highlighted in **b**. Also note in **a** the more open nature of the flap within the TreND complex. **b,** As in **a,** the deAMPylation complex and TreND complexes are aligned via FICD. In addition, the respective BiP($\ell_{7,8}$) regions are also shown. All hydrogen bonds between BiP($\ell_{7,8}$) and FICD are shown. Note, engagement of the FICD flap with the Thr518 region of BiP($\ell_{7,8}$) as an extended β-sheet is only apparent in the deAMPylation complex and the $FICD^{E404C}$ mutation introduced into the TReND complex, eliminates a contact between FICD's Glu404 and Lys516 (within BiP($\ell_{7,8}$)), observed in the deAMPylation complex. **c,** A comparison of both protein complexes (aligned via BiP). Despite considerable displacement in the region of the FICD catalytic domain, flexibility in FICD's linker helix permits both TPR domains to engage BiP in a nearly identical fashion. **d,** An orthogonal view to that presented in **c**. The highlighted FICD catalytic domain-BiP(SBDβ) interface (blue dashed circle) is considerably smaller in the covalent TreND complex (with a protein-protein interface of 298 $Å^2$ versus 671 $Å^2$). The physiological relevance of this diminished and covalently trapped post-catalytic interface remains to be determined. **e,** Residues predicted to be involved in the FICD•BiP complex and mutated[1] are highlighted (green and brown) within the context of the deAMPylation complex. Note, residues found not to affect the rate of BiP AMPylation do not form intermolecular contacts and vice versa. In **b** and **e** hydrogen bonds and hydrophobic interactions are represented by pink and blue dashed lines, respectively.

	Input model	hFICD•dBiP-AMP	dFICD•hBiP-AMP	Best-fit (dimer constrained)	Best-fit (dimer unconstrained)
M_W (kDa)	220	220 ± 10	250 ± 30		
R_m [R_g of complex] (Å)	60.1	58 ± 19 (43 to 70)	63 ± 14 (58 to 68)	57.8	59.7
R_g of FICDs (Å)	34.5	41 (17 to 55)	38 (14 to 52)	36.9	37.2
R_g of BiPs (Å)	69.0	63 (52 to 73)	74 (69 to 79)	66.3	69.0
χ^2 (reduced data)	2.4 ± 2			2.4 ± 0.8	1.7 ± 0.4

Supplementary Table 1. SANS data summary. Biophysical parameters derived from forward scattering (molecular weight) and Stuhrmann analysis of the contrast variation SANS data over the low-q (Guinier) region are shown. The molecular weight (M_W) is estimated by comparison of experimental and theoretical $I(0)/c$ values (the latter assuming an amino acid composition of a 1:1 complex of FICD and BiP-AMP and taking into account buffer component contributions). The mean M_W ± standard deviation (SD) is calculated across all curves excluding the 60% D_2O datasets, which are close to the contrast match points for both partially deuterated complexes. Radii of gyration parameters are best fit or interpolated values ± SE (and/or 95% CI) from the Stuhrmann curve fittings (**Fig. 2d**). Parameters from the best flex-fit heterotetramer models are shown with their theoretical R_g values and overall χ^2 goodness of fit (mean ± SD) against the reduced scattering dataset (**Fig. 2e** and **Supplementary Fig. 3c**). The χ^2 of the input model, against the reduced scattering dataset, is also shown for reference (**Fig. 2a** and **Supplementary Fig. 3c**).

	FICD	$FICD^{L258D}$	GST-FICD[d]
**k_{cat}/K_M (s^{-1} M^{-1})[a]**	630 ± 50	340 ± 30	600 ± 100
**k_{cat} (× 10^{-3} s^{-1})[b]**	10 ± 1	5.7 ± 0.4	9.9 ± 0.9
**K_M (μM)[c]**	17 ± 2	16 ± 2	16 ± 3

Supplementary Table 2. Summary of enzyme kinetic parameters of FICD-mediated BiP deAMPylation. All values are presented as mean ± SEM and are derived from experiments utilising BiP-AMP(FAM) as an indicator of the amount of remaining AMPylated substrate and FICD residues 104–445. [a]Values are taken from **Fig. 3c**. [b]Values are taken from **Fig. 7c**. [c]Calculated based on [a] and [b]. [d]These experimentally derived parameters are from a Michaelis-Menten analysis of GST-tagged FICD (residues 45–458) conducted in[2].

ID	Plasmid name	Description	Encoded protein	Figure	Purification	PMID
**UK1983**	yUlp1(402-621)-StrepII_pET24a	Bacterial expression of yeast Ulp1 protease with a C-terminal StrepII tag	Ulp1-StrepII			31531998
**UK1479**	hsHYPE_45-458_E234G_pGV67	Bacterial expression of hyperactive human GST-TEV-HYPE_45-458	GST-FICD$^{\text{E234G}}$			26673894
**UK2090**	haBiP_27–549_T229A_V461F_pQE30_pSmt3	Bacterial expression of ATPase and substrate binding deficient, lid truncated BiP (N-terminal H6-Smt3 fusion)	Lid truncated BiP$^{\text{T229A-V461F}}$	1	RQ, S75pg	33295873
**UK2093**	hsHYPE_104–445_L258D_H363A_pSmt3_pET28b	Bacterial expression of inactive and monomeric human H363A mutant of His6-Smt3-HYPE(104–445)	FICD$^{\text{L258D-H363A}}$	1, 3a–b, 4a	RQ, S75pg	31531998
**UK2521**	haBiP_27–635_T229A_V461F_pSmt3_pET28b	Bacterial expression of FL ATPase and substrate binding deficient His6-Smt3-BiP(27–635)	BiP$^{\text{T229A-V461F}}$	2	RQ, S75pg	
**UK1954**	hsHYPE_104–445_H363A_pSmt3_pET28b	Bacterial expression of inactive human H363A mutant of His6-Smt3-HYPE(104–445)	FICD$^{\text{H363A}}$	2, 3a–b, 4d	RQ, S200pg	
**UK1801**	EcBirA_WT_pGEX_TEV	Bacterial expression of fastidious E. coli BirA biotin ligase (R118 intact)	GST-BirA			31531998
**UK2359**	haBiP_27–635_T229A_V461F_pQE30_Smt3_Avi	FL hamster BiP ATPase dead, substrate binding deficient with N-terminal H6-SUMO-AviTag and small GS linker	Biotinylated-BiP$^{\text{T229A-V461F}}$	3, 4	MQ	
**UK2054**	hsHYPE_138-445_H363A_pSmt3_pET28b	Bacterial expression of H363A ΔTPR1 mutant of human His6-Smt3-HYPE(138-445)	FICD(ΔTPR1)$^{\text{H363A}}$	3a,	MQ, S200Incr	
**UK2051**	hsFICD_104–186_pSUMO(M3)	Bacterial expression of human FICD/HYPE TPR domain, residues 104–186	TPR Domain	3a, 4b–c	RQ, S75Incr	
**UK2607**	hsHYPE_104–445_E105R_L258D_H363A_pSmt3_pET28b	Bacterial expression of inactive human L258D H363A mutant of His6-Smt3-HYPE_104–445 with TPR mutation	FICD$^{\text{E105R-L258D-H363A}}$	3b, 4d	RQ, S75Incr	
**UK2617**	hsHYPE_104–445_K124E_H131A_L258D_H363A_pSmt3_pET28b	Bacterial expression of inactive human L258D H363A mutant of His6-Smt3-HYPE_104–445 with 2x TPR mutation	FICD$^{\text{K124E-L258D-H363A}}$	3b, 4d	RQ, S75Incr	
**UK2610**	hsHYPE_104–445_H131A_L258D_H363A_pSmt3_pET28b	Bacterial expression of inactive human L258D H363A mutant of His6-Smt3-HYPE_104–445 with TPR mutation	FICD$^{\text{H131A-L258D-H363A}}$	3b, 4d	RQ, S75Incr	
**UK2617**	hsHYPE_104–445_K124E_H131A_L258D_H363A_pSmt3_pET28b	Bacterial expression of inactive human L258D H363A mutant of His6-Smt3-HYPE_104–445 with 2x TPR mutation	FICD$^{\text{K124E-H131A-L258D-H363A}}$	3b, 4d	RQ, S75Incr	
**UK2612**	hsHYPE_104_445_D160C_T183C_L258D_H363A_C421S_pSmt3_pET28b	Bacterial expression of inactive human L258D H363A mutant of His6-Smt3-HYPE_104–445 with TPR stapleable cysteines	FICD$^{\text{L258D-H363A}}$(TPRox) [$_{\text{s-s}}$ FICD$^{\text{D160C-T183C-L258D-H363A-C421S}}$]	3b, 4d	CQ, RQ, S75Incr	
**UK2579**	hsHYPE_104–445_E105R_H363A_pSmt3_pET28b	Bacterial expression of inactive human H363A mutant of His6-Smt3-HYPE_104–445 with TPR mutation	FICD$^{\text{E105R-H363A}}$	3b, 4d	RQ, S200Incr	
**UK2582**	hsHYPE_104–445_K124E_H363A_pSmt3_pET28b	Bacterial expression of inactive human H363A mutant of His6-Smt3-HYPE_104–445 with TPR mutation	FICD$^{\text{K124E-H363A}}$	3b, 4d	RQ, S200Incr	
**UK2583**	hsHYPE_104–445_H131A_H363A_pSmt3_pET28b	Bacterial expression of inactive human H363A mutant of His6-Smt3-HYPE_104–445 with TPR mutation	FICD$^{\text{H131A-H363A}}$	3b, 4d	RQ, S200Incr	
**UK2675**	hsHYPE_104–445_K124E_H131A_H363A_pSmt3_pET28b	Bacterial expression of inactive human H363A mutant of His6-Smt3-HYPE_104–445 with 2 TPR mutations	FICD$^{\text{K124E-H131A-H363A}}$	3b, 4d	RQ, S200Incr	
**UK2296**	hsHYPE_104_445_D160C_T183C_H363A_C421S_pSmt3_pET28b	Dimeric FICD(H363A) made cysteine free apart from disulphide stapleable TPRs	FICD$^{\text{H363A}}$(TPRox) [$_{\text{s-s}}$FICD$^{\text{D160C-T183C-H363A-C421S}}$]	3b, 4d	CQ, RQ, S200Incr	
**UK2091**	hsHYPE_104–445_L258D_pSmt3_pET28b	Bacterial expression of monomeric His6-Smt3-HYPE_104–445. Enzymatically active.	FICD$^{\text{L258D}}$	3c, 5a, 7c	RQ, S75pg	31531998

**UK2760**	hsHYPE_104–445_K124E_L258D_ pSmt3_pET28b	Monomeric His6-Smt3-HYPE_104–445 TPR mutant, bacterial expression. Enzymatically active.	$FICD^{K124E\text{-}L258D}$	3c, 5a	RQ, S75Incr	
**UK2761**	hsHYPE_104–445_H131A_L258D_ pSmt3_pET28b	Monomeric His6-Smt3-HYPE_104–445 TPR mutant, bacterial expression. Enzymatically active.	$FICD^{H131A\text{-}L258D}$	3c, 5a	RQ, S75Incr	
**UK2759**	hsHYPE_104–445_K124E_H131A_ L258D_pSmt3_pET28b	Monomeric His6-Smt3-HYPE_104–445 TPR double mutant, bacterial expression. Enzymatically active.	$FICD^{K124E\text{-}H131A\text{-}L258D}$	3c, 5a	RQ, S75Incr	
**UK2762**	hsHYPE_138–445_L258D_pSmt3_ pET28b	Monomeric ΔTPR1 His6-Smt3-HYPE(138–445), bacterial expression. Enzymatically active.	$FICD(\Delta TPR1)^{L258D}$	3c, 5a	RQ, S75Incr	
**UK2052**	hsHYPE_104–445_pSmt3_pET28b	Bacterial expression of wild type His6-Smt3-HYPE(104–445)	FICD	3c, 5a, 7c	RQ, S200pg	31531998
**UK2763**	hsHYPE_104_445_D160C_T183C_ C421S_pSmt3_pET28b	FICD bacterial expression. TPR stapleable. Enzymatically active.	FICD(TPRox) $[_{s\text{-}s}FICD^{D160C\text{-}T183C\text{-}C421S}]$	3c, 5a	CQ, RQ, S75Incr	
**UK2764**	hsHYPE_104_445_D160C_T183C_ L258D_C421S_ pSmt3_pET28b:	Monomeric FICD bacterial expression. TPR stapleable. Enzymatically active.	$FICD^{L258D}$(TPRox) $[_{s\text{-}s}FICD^{D160C\text{-}T183C\text{-}L258D\text{-}C421S}]$	3c, 5a	CQ, RQ, S75Incr	
**UK2269**	hsHYPE_104–445_A252C_H363A_ C421S_pSmt3_pET28b	Catalytically dead and constitutively dimeric (disulphide stapleable dimer interface) FICD. Trap for BiP-AMP.	Trap $[_{s\text{-}s}FICD^{A252C\text{-}H363A\text{-}C421S}]$	5a	RQ, S200pg	31531998
**UK1314**	pCEFL_mCherry_3XFLAG_C	Mammalian expression of C-terminally 3xFLAG. Neomycin-resistance replaced by mCherry (under SV40 promoter control)	mCherry	5b–c		25858979
**UK1397**	hsHYPE_WT_pCEFL_mCherry	FL CDS of WT HYPE in pCEFL marked with mCherry	FICD	5b–c		26673894
**UK2139**	hsHYPE_E234G_L258D_pCEFL_ mCherry	Mammalian expression of full-length human FICD with E234G and L258D mutations in pCEFL marked with mCherry	$FICD^{E234G\text{-}L258D}$	5b–c		31531998
**UK2676**	hsHYPE_E105R_E234G_L258D_ pCEFL_mCherry	Mammalian expression of FL CDS of L258D E234G HYPE plus TPR mutation in pCEFL marked with mCherry	$FICD^{E105R\text{-}E234G\text{-}L258D}$	5b–c		
**UK2677**	hsHYPE_K124E_E234G_L258D_ pCEFL_mCherry	Mammalian expression of FL CDS of L258D E234G HYPE plus TPR mutation in pCEFL marked with mCherry	$FICD^{K124E\text{-}E234G\text{-}L258D}$	5b–c		
**UK2678**	hsHYPE_H131A_E234G_L258D_ pCEFL_mCherry	Mammalian expression of FL CDS of L258D E234G HYPE plus TPR mutation in pCEFL marked with mCherry	$FICD^{H131A\text{-}E234G\text{-}L258D}$	5b–c		
**UK2679**	hsHYPE_K124E_H131A_E234G_ L258D_pCEFL_mCherry	Mammalian expression of FL CDS of L258D E234G HYPE plus 2 TPR mutations in pCEFL marked with mCherry	$FICD^{K124E\text{-}H131A\text{-}E234G\text{-}L258D}$	5b–c		

Supplementary Table 3. List of plasmids used in the study. *ID* denotes the unique lab identification (UK) number of each plasmid. *Purification* contains information pertaining to any FPLC columns used in the purification of the protein (following strep-tactin, GSH-Sepharose, Ni-NTA affinity chromatography, and on bead cleavage and elution, as appropriate). CQ, HiTrap 5 ml capto Q; RQ, RESOURCE Q 6 ml; MQ, Mono Q 5/50 GL; S75pg, HiLoad 16/60 Superdex 75 prep grade; S200pg, HiLoad 16/60 Superdex 200 prep grade; S75Incr, S75 Increase 10/300 GL; S200Incr, S200 Increase 10/300 GL. *PMID* specifies references to previous publications in which the relevant plasmid has been used, if applicable. FL; full-length.

ID	Primer name	Sequence
**1857**	hsHYPE_BamH1_138S	agcatg *GGATCC* GACTTCGTGGACGCGCTCACC
**1886**	hsHYPE_186_HD3r	aggtgg*AAGCTT*A AAGAGGCAGTGTCCGATCGC
**2194**	hsHYPE_D160C_Fw	AAGGACATCATCCAGGCGtgCTACTTGTACACCAGAGC
**2195**	hsHYPE_D160C_Rv	GCTCTGGTGTACAAGTAGcaCGCCTGGATGATGTCCTT
**2196**	hsHYPE_T183C_Fw	GGTCAACCGCGATCGGtgcCTGCCTCTTGTGGAAG
**2197**	hsHYPE_T183C_Rv	CTTCCACAAGAGGCAGgcaCCGATCGCGGTTGACC
**2263**	haBiP_SUMO_InsAvi_BamHI_Fw	agcgcagaaaattgaatggcatgaaGGATCCGGCACGGTGGTC
**2264**	haBiP_SUMO_InsAvi_BamHI_Rv	tcaaaaatatcgttcaggcccgagccACCAATCTGTTCTCTGTGAGCCTC
**2620**	hsFICD_E105R_Fw	TGGATCCTTGagaGCCAGAGCTG
**2621**	hsFICD_E105R_Rv	CCAATCTGTTCTCTGTGAG
**2626**	hsFICD_K124E_Fw	CAAGCGGGAAgAAGCCCAAAAG
**2627**	hsFICD_K124E_Rv	CCCTGGCGCTTCATCTCC
**2628**	hsFICD_H131A_Fw	GCTCTTCATGgcCGCCCTCAAGATG
**2629**	hsFICD_H131A_Rv	TTTTGGGCTTTTTCCCGC
**2630**	hsFICD_K134A_Fw	GCACGCCCTCgcGATGGACCCG
**2631**	hsFICD_K134A_Rv	ATGAAGAGCTTTTGGGCTTTTTC
**2744**	hsFICD_FL_E105R_Fw	GGGTAAGTTGagaGCCAGAGCTGC
**2745**	hsFICD_FL_E105R_Rv	GCTGGAGAGGCCTTGGTC
**2746**	hsFICD_K124E_FL_Fw	CAAGCGGGAAgAAGCCCAAAAGC
**2747**	hsFICD_K124E_QC_Fw	CGCCAGGGCAAGCGGGAAGAAGCCCAAAA
**2748**	hsFICD_K124E_QC_Rv	TTTTGGGCTTCTTCCCGCTTGCCCTGGCG
**2749**	hsFICD_H131A_QC_Fw	CCCAAAAGCTCTTCATGGCCGCCCTCAAGATGGACC
**2750**	hsFICD_H131A_QC_Rv	GGTCCATCTTGAGGGCGGCCATGAAGAGCTTTTGGG

Supplementary Table 4. List of primers used in the study. *ID* denotes the unique lab identification number of each primer.

Supplementary Note 1: The nature of the immobilised BiP used for BLI binding analyses.

In the BLI experiment shown, we immobilised (in parallel) identical Avi-tagged $\text{BiP}^{\text{T229A-V461F}}$ samples on identical BLI probes and exposed them in parallel to identical solutions of MgATP. This was followed by a brief (50 second) paralleled immersion of the probes into the same solution lacking ATP. At the conclusion of these preparatory steps — which occur before the commencement of the experimental traces shown in **Fig. 3–4** — the samples are still indistinguishable (see **Supplementary Fig. 4d**). Moreover, they remain (functionally) indistinguishable regardless of how much ATP had been hydrolysed and regardless of how much or little ATP hydrolysis has led to domain undocking of the immobilised BiP. Thus, the differences observed in the BLI traces upon these equivalent BiP ligand preparations being confronted by diverse analytes (i.e., FICD mutants in presence or absence of ATP) reflect differences in the BiP-FICD interaction wrought by features of FICD: mutations in FICD, fixation of their TPR domains etc.

Given evidence that it is the ATP-bound domain docked BiP that engages FICD, one might legitimately ask: How plausible is it to imagine that Avi-tagged $\text{BiP}^{\text{T229A-V461F}}$ remains in this state, given reasonable assumptions of rates of ATP hydrolysis, timelines of the experiment and the temperatures involved?

We began by measuring the rate of ATP hydrolysis by the Avi-tagged mutant $\text{BiP}^{\text{T229A-V461F}}$ at 30 ˚C: $k_{\text{cat}} \sim 7.2 \times 10^{-3}\ \text{min}^{-1}$].

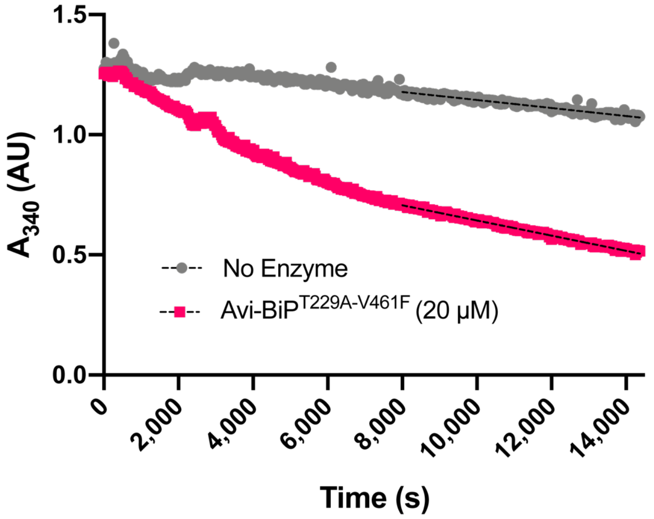

Shown here is a multi-turnover ATPase assay (based on a NADH oxidation-coupled ATP-regenerating system as described in[4], with a total ATP concentration of 5 mM). Each molecule of ATP hydrolysed and subsequently regenerated, results in the oxidation of a molecule of NADH (which in turn results in a reduction in absorbance at 340 nm, A_{340}). Based on the known ε_{340} of NADH, subtracting the spontaneous rate of NADH degradation at 30 ˚C, a BiP-mediated ATP hydrolysis turnover number can be calculated. See above.

If we now make the unfavourable assumption that the rate of ATP-hydrolysis of the N-terminally immobilised Avi-tagged $\text{BiP}^{\text{T229A-V461F}}$ at 30 °C (in the BLI assays presented here and previously[3]) to be even greater and up to four-fold more than the rate of $\text{BiP}^{\text{T229A}}$ ATP-hydrolysis measured at 20 °C in reference[5] [$k_{\text{cat}} \leq 1.0 \times 10^{-2}\ \text{min}^{-1}$], at t = 0 (i.e. the start of the association step which immediately follows the 50 second baseline step in a buffer lacking ATP), less than 1% of the BiP molecules are likely to have hydrolysed their bound ATP (see **Supplementary Fig. 4d**). Even after the 50 second baseline step and the entire cycle of FICD binding and dissociation in buffers lacking ATP (a 1,350 second duration), the extent of ATP hydrolysis of the (initially) ATP-bound, immobilised BiP is expected to be less than 20%. Moreover, binding of FICD to $\text{BiP}^{\text{T229A}}$ does not detectably modulate its intrinsic ATP hydrolysis rate: In Fig. 2B from[3] the presence of 2 µM FICD was observed to not detectably increase the amount of ATP hydrolysed into ADP by 5 µM $\text{BiP}^{\text{T229A}}$, over the course of 2 h incubation at 30 °C. Thus, it seems reasonable to conclude that ≥ 80% of the immobilised Avi-

tagged $BiP^{T229A-V461F}$ in the sample remains ATP-bound and domain docked. This is entirely consistent with its ability to interact with FICD in the mode revealed by the crystals and with the sensitivity of the interaction to mutations in FICD that are predicted to compromise the interaction, as revealed by our experiments. This is also consistent with previous observations that incubation of N-terminally immobilised $BiP^{T229A-V461F}$:ATP in a nucleotide-free buffer for 1,500 s did not result in a significant degree of domain undocking (Fig. EV6A from[3]).

Supplementary References

1. Fauser, J. *et al.* Specificity of AMPylation of the human chaperone BiP is mediated by TPR motifs of FICD. *Nat. Commun.* **12**, 2426 (2021).
2. Preissler, S., Rato, C., Perera, L., Saudek, V. & Ron, D. FICD acts bifunctionally to AMPylate and de-AMPylate the endoplasmic reticulum chaperone BiP. *Nat. Struct. Mol. Biol.* **24**, 23–29 (2017).
3. Perera, L. A. *et al.* An oligomeric state-dependent switch in the ER enzyme FICD regulates AMPylation and deAMPylation of BiP. *EMBO J.* **38**, e102177 (2019).
4. Preissler, S. *et al.* Calcium depletion challenges endoplasmic reticulum proteostasis by destabilising BiP-substrate complexes. *Elife* **9**, 2020.11.03.366484 (2020).
5. Yang, J., Nune, M., Zong, Y., Zhou, L. & Liu, Q. Close and Allosteric Opening of the Polypeptide-Binding Site in a Human Hsp70 Chaperone BiP. *Structure* **23**, 2191–2203 (2015).
