# Supplementary material for: Structures of a deAMPylation complex rationalise the switch between antagonistic catalytic activities of FICD
Source: Nat Commun. 2021 Aug 18;12:5004. doi: 10.1038/s41467-021-25076-7 (PMC8373988; doi:10.1038/s41467-021-25076-7)
Supplement: Supplementary file 3 — Description of Additional Supplementary Files [file 41467_2021_25076_MOESM3_ESM.pdf]

Description of Additional Supplementary Files

File Name: Supplementary Movie 1

Description: Global architecture of the eukaryotic deAMPylation complex. The similarity of the state 1 and state 2 heterodimeric deAMPylation crystal structures of FICD•BiP-AMP is apparent, as is the similarity of the bound BiP-AMP molecule with the isolated structure of BiP:ATP. In the frames that follow we have modelled a heterotetrameric deAMPylation complex by imposing the symmetry of dimeric FICD, and the lid structure of full-length BiP:ATP. Note, the model is compatible with the membrane topology of FICD and is supported by SANS data. Morphs and alignments with the best-fit solution structure (derived from flex-fit SANS analysis with no constraints placed on the FICD dimer interface) appear next. These are suggestive of the existence of increased deAMPylation complex flexibility in solution, relative to the situation in crystallo, especially in the disposition of the BiP SBDα and NBD and to a lesser extent in the FICD TPR domain.

File Name: Supplementary Movie 2

Description: FICD recognises the Hsp70 ATP-state of BiP. Alignment of BiP:ADP with the NBD of BiP-AMP from the heterodimeric deAMPylation complex are depicted. Selected interacting residue pairs in the deAMPylation complex are shown and labelled with hydrogen bonds (pink dashed lines) and representative hydrophobic contacts (blue dashed lines) where appropriate. The same BiP residues in BiP:ADP are also illustrated, highlighting the disruption of NBD-linker interface that occurs in the BiP ADP-state and the resulting incompatibility of BiP:ADP interaction with FICD(TPR). The frames that follow superimpose BiP:ADP and BiP-AMP via their SBDβs. This structural alignment serves to highlight the inaccessibility of BiP:ADP(Thr518) for FICD engagement and AMPylation.

File Name: Supplementary Movie 3

Description: DeAMPylation competency is modulated by Glu234 flexibility. Polder OMIT maps covering catalytically important residues and regions within the active site of both the state 1 (deAMPylation competent) and state 2 (deAMPylation incompetent) complexes are shown. In later frames, superposition of the two structures highlights the lack of a stably positioned catalytic water molecule directly in-line for nucleophilic attack into the Pα-Oγ(Thr518) phosphodiester bond in the state 2 complex. This is likely a result of the altered Glu234 conformation in the state 2 complex causing a shift in the position of the Mg^{2+} coordination complex. Hydrogen bonds are annotated as pink dashed lines.
