# Supplementary material for: Structures of a deAMPylation complex rationalise the switch between antagonistic catalytic activities of FICD
Source: Nat Commun. 2021 Aug 18;12:5004. doi: 10.1038/s41467-021-25076-7 (PMC8373988; doi:10.1038/s41467-021-25076-7)
Supplement: Supplementary file 7 — Reporting summary [file 41467_2021_25076_MOESM7_ESM.pdf]

nature research

Corresponding author(s): Luke A. Perera and David Ron

Last updated by author(s): Jun 28, 2021

Reporting Summary

Nature Research wishes to improve the reproducibility of the work that we publish. This form provides structure for consistency and transparency in reporting. For further information on Nature Research policies, see our Editorial Policies and the Editorial Policy Checklist.

Statistics

For all statistical analyses, confirm that the following items are present in the figure legend, table legend, main text, or Methods section.

n/a	Confirmed	
☐	☒	The exact sample size (*n*) for each experimental group/condition, given as a discrete number and unit of measurement
☐	☒	A statement on whether measurements were taken from distinct samples or whether the same sample was measured repeatedly
☐	☒	The statistical test(s) used AND whether they are one- or two-sided<br>*Only common tests should be described solely by name; describe more complex techniques in the Methods section.*
☒	☐	A description of all covariates tested
☐	☒	A description of any assumptions or corrections, such as tests of normality and adjustment for multiple comparisons
☐	☒	A full description of the statistical parameters including central tendency (e.g. means) or other basic estimates (e.g. regression coefficient) AND variation (e.g. standard deviation) or associated estimates of uncertainty (e.g. confidence intervals)
☐	☒	For null hypothesis testing, the test statistic (e.g. *F*, *t*, *r*) with confidence intervals, effect sizes, degrees of freedom and *P* value noted<br>*Give P values as exact values whenever suitable.*
☒	☐	For Bayesian analysis, information on the choice of priors and Markov chain Monte Carlo settings
☒	☐	For hierarchical and complex designs, identification of the appropriate level for tests and full reporting of outcomes
☒	☐	Estimates of effect sizes (e.g. Cohen's *d*, Pearson's *r*), indicating how they were calculated

Our web collection on statistics for biologists contains articles on many of the points above.

Software and code

Policy information about availability of computer code

Data collection	X-ray crystallography diffraction data was collected using the Diamond Light Source developed Generic Data Acquisition (GDA) software (v9.2).<br><br>SANS data acquisition and instrument control was conducted through the ILL developed NOMAD software.<br><br>Flow cytometry data were acquired using FACSDiva (v8.0.1, BD Bioscience).
Data analysis	SANS data were processed and analysed using GRASP (Graphical Reduction and Analysis SANS Program for Matlab; developed by Charles Dewhurst, ILL); Igor Pro software (v7.08, WaveMetrics) using SANS macros; PEPSI-SANS (v2.8); AnAnaS (v0.9), MULCh and SASSIE contrast calculator.<br><br>Statistical analyses were conducted using Prism (v8.4, GraphPad).<br><br>Immunoblot band quantification was carried out with Image Studio Lite (v5.2.5, LI-COR).<br><br>Crystallography data were processed using DIALS or the xia2 pipeline. Data were further analysed by Pointless (for space group determination) and Aimless (for scaling and merging), both part of the CCP4 module Aimless (CCP4i2 [v1.0.2]). Structures were solved by molecular replacement using the CCP4 module Phaser. Manual model building was carried out in COOT (v0.8.9.2) and refined using refmac5. Metal binding sites were validated using the CheckMyMetal server. Polder (OMIT) maps were generated using the Polder Map module of Phenix (v1.19.2). Structural figures were prepared using UCSF Chimera (v1.15) and PDB structures with evolutionary conservation score depictions were taken from the ConSurf Database. Estimates of interaction surface areas were derived using PDBe PISA (v1.52). Interaction maps were based on an initial output from LigPlot+ (v2.2).

Flow cytometry data were analysed using FlowJo X (v10.0.7r2, BD Bioscience)

For manuscripts utilizing custom algorithms or software that are central to the research but not yet described in published literature, software must be made available to editors and reviewers. We strongly encourage code deposition in a community repository (e.g. GitHub). See the Nature Research guidelines for submitting code & software for further information.

Data

Policy information about availability of data

All manuscripts must include a data availability statement. This statement should provide the following information, where applicable:
- Accession codes, unique identifiers, or web links for publicly available datasets
- A list of figures that have associated raw data
- A description of any restrictions on data availability

The deAMPylation complex crystal structures of monomeric FICD and AMPylated BiP have been deposited in the Protein Data Bank (PDB) with the following accession codes: 7B7Z [https://www.rcsb.org/structure/7B7Z] (state 1) and 7B80 [https://www.rcsb.org/structure/7B80] (state 2). Crystal structure data from previous studies are also available in the PDB, deposited with the following accession codes: 5O4P [https://www.rcsb.org/structure/5O4P], 4U0U [https://www.rcsb.org/structure/4U0U], 5E84 [https://www.rcsb.org/structure/5E84], 7A4U [https://www.rcsb.org/structure/7A4U], 6I7K [https://www.rcsb.org/structure/6I7K], 6I7L [https://www.rcsb.org/structure/6I7L] and 6ZMD [https://www.rcsb.org/structure/6ZMD]. Raw SANS data is available from doi:10.5291/ILL-DATA.8-03-96370. Source data are provided with this paper.

Field-specific reporting

Please select the one below that is the best fit for your research. If you are not sure, read the appropriate sections before making your selection.

- [x] Life sciences
- [ ] Behavioural & social sciences
- [ ] Ecological, evolutionary & environmental sciences

For a reference copy of the document with all sections, see nature.com/documents/nr-reporting-summary-flat.pdf

Life sciences study design

All studies must disclose on these points even when the disclosure is negative.

Sample size	For the majority of independent repeats protein binding or enzymatic activity assays were conducted with a sample size of 1. The reproducibility/robusteness of any observed differences between samples was then confirmed through independent repeats (see below). For the FACS experiments 20,000 live singlets were collected per sample. This number of cells is known to produce a signal-to-noise ratio (in the fluorescent ER-stress reporter) which is more than sufficient to glean variations (if present) between different cell populations.
Data exclusions	Parallel analyses of model goodness of fit to SANS scattering data were conducted with and without the scattering data derived from the sample of dFICD•hBiP-AMP in 100% D2O buffer. The choice to conduct the analysis following the exclusion of this data ('reduced dataset') was made on the basis that all heterotetramer deAMPylation complex models appeared to fit this one scattering curve anomalously poorly (relative to all other scattering curves). Exclusion criteria were not pre-established.
Replication	A number of independent replicates were conducted to verify reproducibility of experimental findings. The number of independent repeats is indicated in the relevant figure legends.
Randomization	Randomization is inherent to the experimental design, and needs no specific comment. For example, cells are grown as a large batch and randomly distributed to the various manipulations (e.g., different transfections or drug treatments), by dilution and aliquoting. Note, that by comparison few if any human drug trials or animal studies ever approach this level of randomization.
Blinding	Blinding was not carried out as the vast majority of experiments were carried out by a single investigator.

Reporting for specific materials, systems and methods

We require information from authors about some types of materials, experimental systems and methods used in many studies. Here, indicate whether each material, system or method listed is relevant to your study. If you are not sure if a list item applies to your research, read the appropriate section before selecting a response.

Materials & experimental systems

n/a	Involved in the study	
[ ]	[x]	Antibodies
[ ]	[x]	Eukaryotic cell lines
[x]	[ ]	Palaeontology and archaeology
[x]	[ ]	Animals and other organisms
[x]	[ ]	Human research participants
[x]	[ ]	Clinical data
[x]	[ ]	Dual use research of concern

Methods

n/a	Involved in the study	
[x]	[ ]	ChIP-seq
[ ]	[x]	Flow cytometry
[x]	[ ]	MRI-based neuroimaging

Antibodies

Antibodies used	Primary antibodies against hamster BiP (chicken anti-BiP), eIF2α (mouse anti-eIF2α) and FICD (chicken anti-FICD). N.B. these antibodies are not available commercially.
Validation	These antibodies were validated in the following publications, respectively:<br>1. Avezov, E. et al. Lifetime imaging of a fluorescent protein sensor reveals surprising stability of ER thiol redox. J. Cell Biol. 201, 337–49 (2013).<br>2. Scorsone, K. A., Panniers, R., Rowlands, A. G. & Henshaw, E. C. Phosphorylation of eukaryotic initiation factor 2 during physiological stresses which affect protein synthesis. J. Biol. Chem. 262, 14538–43 (1987).<br>3. Preissler, S. et al. AMPylation matches BiP activity to client protein load in the endoplasmic reticulum. Elife 4, e12621 (2015).

Eukaryotic cell lines

Policy information about cell lines

Cell line source(s)	CHO-K1 (ATCC, CCL-61); CHO-K1 FICD–/–; CHO-K1 S21; CHO-K1 S21 FICD–/– (N.B. the CHO-K1 derived cell lines are not commercially available).
Authentication	The cells were phenotypically validated as proline auxotrophs and their Cricetulus griseus origin was confirmed by genomic sequencing.
Mycoplasma contamination	Cell lines were subjected to random testing for mycoplasma contamination using the MycoAlert Mycoplasma Detection Kit (Lonza) and tested negative.
Commonly misidentified lines<br>(See ICLAC register)	No commonly misidentified cell lines were used.

Flow Cytometry

Plots

Confirm that:

- [x] The axis labels state the marker and fluorochrome used (e.g. CD4-FITC).
- [x] The axis scales are clearly visible. Include numbers along axes only for bottom left plot of group (a 'group' is an analysis of identical markers).
- [x] All plots are contour plots with outliers or pseudocolor plots.
- [x] A numerical value for number of cells or percentage (with statistics) is provided.

Methodology

Sample preparation	FICD over-expression-dependent induction of unfolded protein response signalling was analysed by transient transfection of CHO-K1 S21 FICD–/– UPR reporter cell lines with plasmid DNA encoding the complete FICD coding sequence (with mutations as indicated) and mCherry as a transfection marker, using Lipofectamine LTX (Thermo Fisher). 0.5 µg DNA was used to transfect cells growing in 12-well plates. 40 h after transfection the cells were washed with PBS and collected in PBS containing 4 mM EDTA before FACS analysis.
Instrument	LSRFortessa cell analyser (BD Biosciences).
Software	Flow cytometry data were collected using FACSDivaA (v8.0.1, BD Bioscience)<br><br>Flow cytometry data were analysed using FlowJo X (v10.0.7r2) and Prism (v8.4, GraphPad).
Cell population abundance	20,000 live single cells were analysed per sample.
Gating strategy	Preliminary gating of live cells was based on FSC-A/SSC-A scatter.<br>Gating for singlets was based on FSC-W/SSC-A scatter.<br>An mCherry+ subset was gated with mCherry FI (561_610/20) ≤ 10,000 RFU.<br>High and low-stress populations (of mCherry+ subset cells) were assigned through the use of a bisector gate in the XBP1::Turquoise FI (405_450/50 nm) at 600 RFU.

- [x] Tick this box to confirm that a figure exemplifying the gating strategy is provided in the Supplementary Information.
